# Designing and Facilitating Collaborative Research Design and Data Analysis Workshops: Lessons Learned in the Healthy Neighborhoods Study

**DOI:** 10.3390/ijerph16030324

**Published:** 2019-01-24

**Authors:** Andrew Binet, Vedette Gavin, Leigh Carroll, Mariana Arcaya

**Affiliations:** 1 Department of Urban Studies and Planning, Massachusetts Institute of Technology, 77 Massachusetts Ave., Cambridge, MA 02139, USA; abinet@mit.edu; 2 Conservation Law Foundation, 62 Summer St., Boston, MA 02110, USA; vgavin@clf.org; 3 SEIU Healthcare Pennsylvania, 1500 N. 2nd St., Harrisburg, PA 17102, USA; leigh.m.carroll@gmail.com

**Keywords:** participatory action research, community engagement, instrument design, data analysis, urban development, community health

## Abstract

One impediment to expanding the prevalence and quality of community-engaged research is a shortage of instructive resources for collaboratively designing research instruments and analyzing data with community members. This article describes how a consortium of community residents, grassroots community organizations, and academic and public institutions implemented collaborative research design and data analysis processes as part of a participatory action research (PAR) study investigating the relationship between neighborhoods and health in the greater Boston area. We report how nine different groups of community residents were engaged in developing a multi-dimensional survey instrument, generating and testing hypotheses, and interpreting descriptive statistics and preliminary findings. We conclude by reflecting on the importance of balancing planned strategies for building and sustaining resident engagement with improvisational facilitation that is responsive to residents’ characteristics, interests and needs in the design and execution of collaborative research design and data analysis processes.

## 1. Introduction

Community-engaged, action-oriented research approaches involve communities that are impacted by the issues being studied. Such approaches include the overlapping traditions of participatory action research (PAR) [1,2], community-based participatory research (CBPR) [3], participatory epidemiology [4], and citizen science [5,6]. The universe of participatory and community-engaged research comprises studies with vastly different levels of community ownership over the research, types of community engagement, and kinds of outputs [7,8,9,10], but is united by the shared perspective that communities most impacted by a phenomenon have unique forms of expertise concerning the phenomenon which should, in turn, be integral to how the phenomenon is understood and ultimately addressed.

PAR, CBPR, and other community-engaged research approaches represent a small—albeit growing—share of social science research, in part because participatory approaches to research can be more time-consuming, financially costly, risky, and challenging to manage than conventional approaches [3,11]. However, these relative disadvantages are more a product of how research has been institutionalized than of problems inherent to the engagement of community members in research. For example, Brown et al. [12] demonstrate that the unfamiliarity of many institutional review boards (IRB) with CBPR can lead to misunderstandings, obstacles, and delays to research that may reduce the benefits of community-engaged research for participants and even potentially cause harm. This is despite the fact that CBPR elevates the core principles of the use of human subjects in research that IRBs seek to uphold. Community-based versus traditional approaches to research also pose special challenges for funders, including: a longer research timeframe, the need for more flexible funding, the need to support multiple intermediary organizations that facilitate crucial community-building, and the fact that traditional research evaluation approaches may be ill-suited to evaluating participatory research [13]. These practical difficulties can be compounded by the fact that institutional and disciplinary cultures exercise significant limits on what types of research and knowledge may be considered “legitimate” [14,15,16]. Finally, community-engaged scholarship can pose a professional risk for researchers employed in academia because it has historically been under-valued and under-rewarded in faculty promotion and tenure decisions [17].

These barriers help explain why the participation of community members within community-engaged research projects varies considerably in terms of depth and scope. For example, a 2003 review of 25 CBPR studies found that while all engaged community members in participant recruitment, only four studies engaged community members in the analysis of data [18]. Some studies involve community members in the design of research instruments, while others may bring community members on board to collect data after instruments have been designed by academic partners [7,19,20]. Some studies engage local organizations and advocates in using research data, while others may only engage individual residents [9,21]. Additionally, the role that community members are invited to play in participatory research can vary based on their personal characteristics: for example, Shamrova and Cummings [22] find that young people tend to be under-included in the research design and analysis phases of CBPR studies, and Vaughn et al. [23] find that immigrants involved in CBPR studies are much less commonly involved in data analysis and interpretation than they are in the recruitment of study participants and the collection of data.

In addition to institutional, cultural and social barriers that prevent full and meaningful engagement of communities in research, a shortage of instructive guidance on how to meaningfully and substantively engage community members in key stages of the research process also hinders further development of PAR, CBPR, and similar approaches. Although scholarly resources exist, most focus on the principles of participatory approaches and challenges researchers may face when employing them [11]. With some notable exceptions [3,24], there are few detailed, step-by-step examples of how research teams actually planned, structured, and implemented community involvement in specific aspects of the research process, and how the principles of community-engaged research are realized in the design features of specific participatory research processes.

In order to add to the nascent set of practical guides on implementing community-engaged research, this article describes the collaborative research design and collaborative data analysis processes implemented by the Healthy Neighborhoods Study (HNS). The HNS is a longitudinal, multi-site study exploring the relationship between urban development and community health in nine rapidly-changing neighborhoods in the Boston metropolitan area [25]. While the HNS integrates community engagement into all phases of research, this article focuses on collaborative instrument design and data analysis. A shortage of guidance on how to collaboratively design data collection instruments and how to collaboratively analyze study data could reflect the fact that community members are often less engaged in instrument design and data analysis than they are in other parts of the research process [7]. However, limited community engagement in instrument design and data analysis could also be caused, in part, by a lack of practical guidance in these areas.

The purpose of this article, therefore, is to detail how the HNS planned, structured, executed and documented community-engaged data collection instrument design and data analysis, and how the outputs from these processes have been used by members of the HNS research consortium. Our goal is to provide one practical example of PAR approaches to these two research phases given that academic journal formatting and length requirements often preclude researchers from publishing the full extent of CBPR/PAR methods [26]. First, we provide context and background on the Healthy Neighborhoods Study. Second, the methods section describes how we developed collaborative research design and collaborative data analysis workshops in the baseline period of the HNS. The results section presents the content of each workshop, describes how the workshops were facilitated in practice, and reviews outcomes of these workshops, which include: a survey instrument which measures previously-validated and newly developed constructs, resident-generated hypotheses and interpretations of findings, and education and capacity-building for community members and grassroots organizations. We conclude by discussing the strengths and limitations of our approach, and lessons learned for engaging communities in research design and data analysis.

## 2. Healthy Neighborhoods Study

The HNS is a longitudinal PAR study investigating the relationship between urban development and community health across nine Boston metropolitan area neighborhoods. The HNS is a project of the HNS Research Consortium (HNSRC), a partnership of residents, grassroots community organizations, academic and non-profit institutions, and state government agencies. The study examines residents’ relationships with changing neighborhood conditions to identify health risks and protective factors that are introduced by new urban development and associated changes in the built and social environment. It also identifies development activities that may effectively address health risks faced by residents. Throughout the research process, local partner organizations and resident researchers are supported by the HNSRC in applying findings to neighborhood-based efforts to improve community health. The nine communities were selected for the study because they are neighborhoods where large investments in urban development are expected to occur in the near term, and which exhibit relatively poor health outcomes and economic disadvantage. The rationale for adopting a PAR approach to the HNS, detailed descriptions of the screening processes for selecting study communities and developing partnerships with local organizations, and baseline findings from the study, have been published by Arcaya et al. [25]. The HNS is now in its third year of data collection. This manuscript focuses on the study’ baseline period (2015–2016) because it was the most intensive period of innovation in developing the HNS’s PAR processes. However, we also include information on the 2018 collaborative data analysis phase to show how those processes have evolved.

The HNS is centered on a network of 45 “resident researchers”, residents of the study communities with varying levels of formal education and research experience, who work in collaboration with academic, non-profit and public agency partners to design the study protocol and data collection tools, collect primary data, analyze primary and secondary data, and act on the data at the local and regional level. Each study community is represented by 4–5 resident researchers who are recruited by the HNS’s local partner organization in that community. Organizations typically recruited residents with whom they had pre-existing relationships, usually through residents’ participation in programs offered by the organization or prior volunteer work for the organization. The only requirements for recruitment were that resident researchers were 16 years of age or older, were residents of the study neighborhood or former residents who had recently moved, had the ability to participate in PAR workshops in English, and had the ability to conduct research in English, Spanish, and/or Haitian Creole. Resident researchers were paid a wage of USD $15 per hour [25].

The HNS PAR process comprises five phases: (1) scoping: building relationships and setting goals and expectations; (2) knowledge exchange and research design; (3) training; (4) data collection; and (5) data analysis. These phases are described in detail in Arcaya et al. [25]. In the baseline period of the study, we completed all five phases in 15 months, from October 2015 to December 2016 (Table 1). Communities began the process at staggered intervals based on the capacity of the facilitation team and the readiness of local partners. In subsequent years of the study, all communities have repeated all five phases at a common pace over 12-month cycles.

## 3. Methods

Below, we describe how the HNS facilitators developed the “collaborative research design” (CRD) and “collaborative data analysis” (CDA) workshops (Table 1, phases 2 and 5). A group of four personnel from lead academic and non-profit partner institutions developed and subsequently facilitated each workshop. The HNS academic investigators found ample guidance in existing literature on how to establish partnerships with community members and local organizations, train community in research ethics and study protocol, and gather data using surveys (Table 1, phases 1, 3 and 4). However, a review of scholarly literature on the mechanics of community-engaged research processes produced fewer resources than expected that could serve as a foundation for developing and facilitating collaborative processes to engage resident researchers in instrument design and data analysis (Table 1, phases 2 and 5). As a result, we designed the workshops that comprised the research design and data analysis phases largely from scratch, incorporating selected activities and ideas from other resources when possible and appropriate.

### 3.1. Developing Collaborative Research Design Workshops (Phase 2)

The facilitators developed the series of collaborative research design workshops between October 2015–January 2016, as we formed partnerships with local organizations and initiated resident researcher recruitment. We had three goals in mind as we developed these workshops: first, to familiarize resident researchers with PAR principles and build the capacity and cohesion of each community’s team of resident researchers; second, to facilitate the development of a survey tool with one core component common to all nine study communities, to which each community would add its own set additional measures based on its own priorities; and third, to reach consensus on a strategy for future qualitative data collection. We began the process of designing the CRD workshops by doing an online scan for resources that either (1) gave detailed guidance on how to design research instruments with community members [27], or (2) contained practical resources that could be adapted for use as components of our workshops [28]. In parallel, we conducted a review of academic literature on PAR and CBPR projects that included descriptions of how instruments were developed in collaboration with community residents [20]. This review identified publications that provided high-level guidance on participatory approaches to research design, which provided the basis for the order and goals of the five CRD workshops. However, few publications provided detailed descriptions of how community engagement in instrument design was actually facilitated in practice. As a result, we developed original resources in the form of facilitation guides for each of the five CRD workshops. These guides integrated some components adapted from outside resources with others developed by the HNS facilitators. Over the course of the five workshops, components included: experiential observation, journaling exercises, storytelling, interactive exercises, and community conversations.

### 3.2. Developing Collaborative Data Analysis Workshops (Phase 5)

During October–November 2016, we prepared facilitation resources for collaborative data analysis (CDA) workshops which were held in November–December 2016. We followed the same process of completing an online search of non-academic resources and scholarly publications, but found little practical guidance in the academic and non-academic PAR and CBPR literature on how to facilitate collaborative analysis of quantitative data for people with no or limited prior data analysis expertise. As a result, the collaborative data analysis workshops almost exclusively comprised components developed by the HNS facilitators, who drew on their knowledge of the HNS and their previous experience with PAR to plan workshops. The goal was to that would generate valuable analytical contributions from the resident researchers including testable hypotheses, analytical models, interpretations of raw data and analytical outputs, and recommendations to improve data collection instruments and processes. To assist us in this process, we held preparatory workshops in November 2016 with resident researchers in four of the nine study communities to generate initial hypotheses with resident researchers in November 2016 that would guide the development of the agenda for three larger tri-community collaborative data analysis workshops that took place a month later. In July 2018, we drew on these materials, notes, and reflections from facilitating the first set of CDA workshops, and feedback from resident researchers and community partner organizations, to design a second collaborative data analysis workshop held in August 2018 shortly after the conclusion our second cycle of data collection (October 2017–June 2018). To prepare for both iterations of the CDA workshops, the academic investigators conducted initial analyses before meeting with resident researchers in order to be able to provide resident researchers with results to review, interpret and respond to. For example, in preparation for the 2018 CDA workshop, academic investigators designed and conducted an exploratory factor analysis inspired a hypothesis proposed by resident researchers in the 2016 CDA workshop about potential latent variables underlying patterns among a set of observed variables measuring one of the study’s constructs. The results of the exploratory factor analysis were presented to resident researchers for interpretation and discussion, during which a collective decision was made about how to conduct a confirmatory factor analysis.

### 3.3. Documentation

We documented our CRD and CDA processes deliberately to facilitate reflection about how the mechanics of these particular processes connect to discussions about the principles, challenges and opportunities of participatory research that prevail in the scholarly literature. Specific documentation methods included: note-taking, photography, audio and video recording, and participant homework exercises. The facilitation team reviewed these materials between workshops and project phases to reflect on our progress and adapt our plans for how to best facilitate resident researcher participation during subsequent stages of the process.

## 4. Results

The knowledge exchange and instrument design phase consisted of five collaborative research design workshops that were each facilitated in sequential order in each of the nine study communities between March–August 2016 (Table 2).

Over the course of seven months, 45 resident researchers and nine community organizers completed the series of workshops, with each resident researcher participating in a total of 15 h of workshops over five sessions. Each team of resident researchers completed the five-workshop series over a period of six to eight weeks, with the intervals between consecutive workshops ranging from three days to more than two weeks, depending on the resident researchers’ availability. The facilitation materials for these five CRD workshops can be found in the Supplementary Materials (S1).

At their first CRD meeting, resident researchers got to know one another, completed a series of team activities to learn about the principles and goals of PAR, and participated in group dialogue exercises designed to draw on their lived experience to develop a list of ideas and themes about how the changes in their neighborhoods were related to issues of health and wellbeing. At the second meeting, facilitators led resident researchers through activities designed to generate research questions to guide inquiry into the themes identified in the first workshop. To prepare for the third workshop, facilitators compiled all themes and questions generated by each group of resident researchers into a master list. During the third workshop, the facilitators introduced resident researchers to different methodologies (e.g., surveys, interviews, focus groups, photovoice) and types of data (e.g., quantitative, qualitative), and then guided resident researchers through a process to select the most important themes and research questions from the master list, identify the data needed to address these themes and questions, and match research methods with the identified data needs. All groups reached consensus that quantitative surveys and qualitative semi-structured interviews would both be necessary, and agreed to develop a quantitative survey in the baseline period, followed by a semi-structured interview tool in the second year of the study. Resident researchers in the earliest three communities to begin the CRD workshops agreed to try to develop a survey comprised of one common component shared across all communities, plus additional community-specific measures based on the issues prioritized by resident researchers representing each community.

To prepare for the fourth workshop, facilitators reviewed the existing literature on themes prioritized by resident researchers to identify an array of previously validated measures that could measure the constructs that resident researchers prioritized during the third workshop. During the fourth workshop, resident researchers selected from among this array the measures that they felt were best suited to measure the constructs they had previously prioritized, and reached consensus on whether to adopt each measure as is or make small modifications to better suit the local context. For example, one group of resident researchers proposed changing the phrase “spray-painting graffiti” from Sampson et al.’s collective efficacy scale to “spray-painting negative words” because they felt that graffiti was considered a form of art by their fellow community members and that the question—intended to measure the respondent’s sense of how much neighbors were likely to intervene in troubling events—would thus be misinterpreted [32]. During the fourth CRD workshop, resident researchers also began to create their own measures for constructs of interest for which no previously validated measures could be found in the existing literature, including a person’s ability to fulfill their most important priorities and a person’s feelings of ownership over neighborhood-level social, economic, and physical changes such as the development of new housing and amenities and the creation of new jobs.

After each group of resident researchers completed the fourth workshop, the facilitators shared their choices and contributions with other groups of resident researchers, who had the opportunity to suggest further edits that would be necessary for them to reach consensus on the inclusion of each measure in the survey, as well as to contribute to the development of new measures. For example, one group of resident researchers proposed developing a new measure for respondents’ feelings of ownership over neighborhood changes. The facilitators then shared this proposal for a new measure of feelings of ownership with two other groups of resident researchers, who then proposed specific types of changes and aspects of “ownership” to measure. These modifications were returned to the initial group of resident researchers, who drafted a series of questions to measure this construct. The facilitators then shared these questions with the other two groups, who tested the questions on friends and family and suggested further modifications based on how well they thought the questions worked.

In the final collaborative research design workshop, resident researchers received information on other groups’ contributions and feedback on their own contributions from their peers in other communities. Then, they made additional modifications to survey components based on input from their peers, and decided which measures to include in the common component of the survey and which measures they wanted to include in their community-specific component. Although our initial goal was that each community would have a survey instrument comprised of one component common to all nine communities, plus additional community-specific components, all groups ended up wanting to include the measures prioritized by other communities and to be able to see patterns across the entire study population to make cross-community comparisons. Thus, we reached consensus with resident researchers and partner organizations to integrate all topics of interest into a unified survey tool to be used across all study sites. Resident researchers across all nine communities agreed to measures across 10 key domains: housing, neighborhood fit, social support, local business, financial security, food security, ability to meet one’s own life priorities, experiences with discrimination, physical health and mental health, and ownership of neighborhood changes. There was only one exception to this consensus, wherein one community chose to add two questions to their survey about stigma related to drug use due to pressing local debates about access to addiction treatment facilities, but other communities declined to incorporate these questions.

Outcomes from conducting the series of five collaborative research design workshops in all nine study communities during 2016 included the study’s Theory of Change, the HNS baseline survey [25], resident researcher onboarding and capacity-building, deeper community organization partnerships, and improved collaboration between resident researchers and community partner organizations and other academic and public-sector members of the HNS consortium. During each subsequent year of the HNS, facilitators have condensed and modified these workshops in order to (1) onboard new resident researchers and familiarize them with the approach and core themes of the HNS, and (2) to refine the survey and develop a unified semi-structured interview tool for a nested longitudinal cohort. An additional 43 workshops derived from the original 2016 CRD workshop materials were facilitated for a total of 52 new and returning resident researchers over 129 h between June–November 2017.

In December 2016, a total of 32 resident researchers participated in the collaborative data analysis workshops (Table 3). First, preparatory workshops were held in four of the nine study communities, according to resident researchers’ availability and capacity, to generate preliminary hypotheses about the relationships between different variables on which the survey gathered data. Second, the facilitators synthesized outputs from these workshops to develop an agenda for one longer workshop which was facilitated three times, each bringing resident researchers and representatives of local partner organizations in three geographically proximate study neighborhoods together. For resident researchers, this was the first opportunity they had to meet and collaborate with their counterparts from other study communities. Outputs from these workshops included: resident researchers’ interpretations of summary statistics and preliminary structural equation modelling and factor analysis outputs based on resident researcher hypotheses generated in the four preparatory workshops; ideas for refining the survey in the following year of the study ideas for themes to explore through secondary data analysis and semi-structured interviews; feedback from resident researchers on their experience in that role during the baseline phase of the study; and increased cross-community collaboration.

In early August 2018, as part of the second iteration of the collaborative data analysis phase, we facilitated one large collaborative data analysis workshop bringing together 23 resident researchers from all nine study communities. Resident researchers participated in full-group activities and then rotated in small groups through four stations representing study themes prioritized by resident researchers for further analysis (Table 4). Similar to the previous CDA workshops, outputs from this CDA workshop included: Resident researcher interpretation of summary statistics and preliminary regression and factor analysis outputs; ideas for refining the survey and semi-structured interview tools; feedback from resident researchers on their experience in that role to date; and increased cross-community collaboration. The facilitation materials for this workshop can be found in the Supplementary Materials (S2).

Although we developed detailed facilitator and participant guides for each CRD and CDA workshop, we never followed them exactly as written. We improvised considerably during workshops, and we also refined and revised materials between workshops in response to the different interests, capabilities and needs of each community of resident researchers. As a result, there was some variation across workshops despite using consistent facilitator and participant guides. Continuous engagement between groups of resident researchers via meetings in person and by phone allowed the facilitators to integrate lessons learned from each workshop into the next, foster relationships with and among resident researchers, and synthesize each community’s ideas, questions and progress in order to clearly communicate these contributions across all nine groups of resident researchers and other HNSRC members.

## 5. Discussion

In this article, we have reported how the HNS planned, structured, executed, and documented collaborative research design and data analysis processes that meaningfully engaged residents of our study communities in these key phases of the research process. We report specifically on how nine different groups of community residents were engaged in developing a shared survey instrument, and how they were involved in generating and testing hypotheses and interpreting descriptive statistics and preliminary findings, processes for which there is a shortage of instructive, step-by-step guidance in the existing literature. Below, we summarize key results and discuss the strengths and limitations of our approach and lessons relevant to other researchers seeking to engage residents of study communities in designing research instruments and analyzing data.

Facilitating collaborative research design required five workshops with community residents and organizational partners in each of the nine HNS study communities, and resulted in a common survey instrument used to collect HNS data across the nine neighborhoods. The 2016 collaborative research design workshops were successful in: (1) introducing a network of 45 resident researchers across nine study communities to participatory action research and the central themes of the Healthy Neighborhoods Study; (2) soliciting resident researchers questions and insights about the relationship between health and development in their neighborhoods; and (3) developing a comprehensive survey tool used by resident researchers to collect data from residents of their neighborhoods. These workshops were condensed and adapted to orient new resident researchers, refine the baseline survey instrument, and develop a semi-structured interview tool between June and November 2017.

In December 2016, we facilitated a collaborative data analysis workshop in each of the three geographic clusters of HNS study communities: north of Boston, central Boston, and south of Boston. In 2018, HNS combined collaborative data analysis activities into one workshop, bringing together resident researchers from all nine HNS study communities. The collaborative data analysis workshops in 2016 and 2018 successfully introduced resident researchers to analytic methods, and elicited hypotheses, interpretations of preliminary analyses, and directions for further research. The CDA workshops also allowed resident researchers to meet and share ideas with their counterparts from more distant communities, as well as for the entire HNS team to celebrate our accomplishments.

Facilitation materials from the 2016 CRD and 2018 CDA workshops have been included as Supplementary Materials (S1, S2) to this article in order to aid other practitioners of community-engaged research.

The HNS is well-suited to the use of a community-engaged research approach like PAR because the study investigates the relationship between two things that people typically know and think about on an everyday basis—their health and their neighborhoods. Focusing on health and neighborhood changes proved to be advantageous during both the collaborative research design and collaborative data analysis workshops because participants were able to draw from experiential and embodied knowledge when making contributions, without requiring significant subject matter training. PAR was effective in soliciting meaningful contributions from community residents, as evidenced by the fact that all of the domains that resident researchers prioritized for investigation to understand the relationship between health and neighborhood development—housing, neighborhood fit, social support, local business, financial security, food security, ability to meet one’s own life priorities, experiences with discrimination, and ownership of neighborhood changes—have subsequently shown associations with physical and/or mental health outcomes in preliminary analyses of HNS survey data [25]. In particular, the strengths of a PAR approach to instrument design were in choosing constructs to measure, adjudicating between different pre-existing validated measures of these constructs and modifying them to fit the local context, and developing new measures for constructs generated by resident researchers for which there was no suitable precedent in the literature.

We offer six lessons from our experience designing and facilitating the HNS’s CRD and CDA workshops that may be of use to other researchers seeking to engage community members in similar processes in other community-based studies, especially those with multiple study sites and/or groups of resident researchers working in parallel.

The first lesson we learned was that maintaining continuity between workshops for each group of resident researchers was more important and challenging than we expected. Groups of resident researchers occasionally experienced gaps of up to four weeks between collaborative research design workshops due to the geographic dispersal of study communities, the fact that facilitators were working with up to nine different teams of resident researchers at once, and the difficulties of reconciling resident researcher and community partner organizations’ busy schedules. As a result, workshop facilitators often spent more time than expected refamiliarizing each group with the progress made to date and helping them reestablish their orientation within the CRD process. More materials, activities and resources—such as journaling assignments or social media interaction—might have helped some resident researchers stay more intellectually and socially connected to the project between workshops.

The second lesson we learned was the need to treat the facilitation process as one of trial and error. As mentioned in our results, we ultimately never followed the facilitation guides for each workshop exactly as written. With each different group of resident researchers, some parts of the process were more effective than others. We learned that with each iteration of a workshop, it was necessary to adapt the facilitation materials and techniques to both reflect our prior experience and align with the particular group of resident researchers and their various sets of interests, concerns and contributions. While some of this adaptation was planned in advance, we also improvised on the workshop materials in real time to be responsive to the needs and priorities of resident researchers.

Third, we learned the importance of having the same small group of four facilitators share the responsibility for facilitating all workshops across all study sites. Continuity of facilitation along the series of five CRD workshops in each specific study community was necessary for ensuring that each session built on those preceding it, and that trusting relationships emerged between facilitators, resident researchers, and representatives of local partner organizations. Similarly, continuity of facilitation across different study communities was necessary for synthesizing the contributions of each community’s group of resident researchers and communicating them to the others, which enabled all groups to provide feedback on one another’s ideas and build on each other’s progress. Finally, consistent facilitation ensured that each community followed a similar process, while also ensuring that the workshops could be tweaked from one community to the next based on their efficacy.

Fourth, we learned that staggering the initiation of the CRD workshops helped us make useful changes to the workshop materials and facilitated more collaboration and consensus across groups of resident researchers than we expected, even though we had only intended to stagger the groups to make workshop administration logistically easier. Staggering allowed the facilitators to pilot the facilitation materials and make important adaptations to the materials when we realized that certain components were ineffective for more than one group. Staggering also allowed the facilitators to ensure that each group was able to build on the contributions of other groups at multiple phases of survey development, which would not have been possible if all communities completed each workshop simultaneously. For example, one goal of the fourth CRD workshop was to generate new measures; communities who began the CRD process earliest used the fourth workshop to draft new measures for constructs for which there were no appropriate precedents in the literature, whereas other communities who began the CRD process later than others used the fourth workshop to practice using these measures on one another to evaluate their utility and make further modifications if necessary. Finally, while our initial objective was to develop a survey that had a core component shared by all nine communities plus additional modules specific to each community’s interests and needs, staggering enabled communities to reflect on the prior contributions of other groups and reach a cumulative consensus to use one unified instrument across all nine study neighborhoods. One notable disadvantage of staggering the CRD process across the nine communities was that it was difficult to arrange cross-community engagements because each group was usually at a different point in the process. Resident researchers have subsequently indicated to the facilitation team that more interaction with other groups of resident researchers would have improved their experience and sense of belonging within the larger HNS consortium.

Fifth, we learned that building trust among resident researchers and between resident researchers and facilitators was crucial to the success of these workshops. Although one of our goals in designing the workshops was to build good working relationships among different members of the HNS team, it was only through actually facilitating the workshops that we learned how important building trust was to fulfilling the other objectives of the workshops, such as selecting topics to investigate with the survey tool. For example, we quickly learned that planning ample time to discuss race, class, gender, and other types of power relations was crucial to building trust between resident researchers and the facilitators. However, conversations about power relations emerged at unpredictable and inconsistent points in the process with each group. Being flexible to talk about power relations when the need arose built trust by showing responsiveness to resident researchers, and made it easier for resident researchers and facilitators to have frank conversations about difficult topics such as discrimination and inequality, which were central to designing our instruments, developing hypotheses, and interpreting preliminary findings.

To facilitate trust between resident researchers and the workshop facilitators, we also learned that it was critical for facilitators to avoid being defensive when resident researchers challenged aspects of the participatory process. Instead, the facilitators learned to welcome these conflicts as learning opportunities. For example, during the initial collaborative research design workshops in 2016, one resident researcher criticized the extent to which HNS facilitators from academic institutions deferred to the experiential intelligence of resident researchers, and argued that the facilitators needed to do a better job of bringing the various forms of expertise that we have to the table and integrating them into the shared process. He later reflected that he only decided to trust the facilitators after we responded curiously, rather than defensively, to these critiques.

Sixth, explicitly discussing how research decisions could impact HNS’s potential as a catalyst for community change increased resident researchers’ engagement in the workshops. Resident researchers were sometimes skeptical about the potential for research to bring about positive changes in their communities. Being transparent about the fact that using research for action was a central principle of our PAR approach, and being specific about pathways for connecting research to action, helped some resident researchers engage more deeply. Ultimately, the value of participatory research comes from authentic collaboration, and achieving authentic collaboration requires that community members be treated as partners who have reasons for wanting to understand and change both the conditions of their communities, and the conditions under which their communities are studied.

We also note three limitations of this account of the HNS’s collaborative research design and data analysis processes. First, we note that the development and facilitation of the CRD and CDA workshops was a largely self-contained process. Although the facilitators received feedback on the design and outcomes of the CRD and CDA processes from members of the HNSRC including state and local public health and urban planning agencies, academic partners, each community partner organization, and our funder, we did not have the opportunity to receive constructive criticism on the design of the CRD and CDA workshops from colleagues outside of the HNSRC. Second, we note that while HNSRC policy is to have a mix of academic and resident researcher authors on all publications of empirical findings from HNS data, resident researchers were not involved in writing this article because they were not involved in the design or facilitation of the CRD or CDA workshops that they participated in. As a consequence, this account is solely that of the facilitation team and not the workshop participants themselves.

The third and final limitation is that we have not yet developed a process to formally evaluate resident researchers’ experiences of being involved in the study, and thus we are unable to provide a systematic report on resident researchers’ experiences of the CRD and CDA processes. Instead, this account has relied solely on the results of the facilitators’ documentation, experiences, and many hours of informal conversation with resident researchers. From informal conversations with resident researchers, we know that their involvement in the CRD and CDA has led some to seek additional responsibilities within the HNS, for example by taking on leadership roles at their study sites or speaking about PAR at conferences alongside HNS academic investigators. Four resident researchers have been interviewed about their experiences for a series of blog posts about PAR [33,34,35,36]. In the future, the HNS facilitators plan to collaborate with resident researchers to develop a process for systematically evaluating their experiences as members of the HNS team.

Other researchers who seek to involve community members in the development of data-gathering instruments and the analysis of the data that they collect may find the HNS’s workshop materials useful. While each PAR project is unique, the materials we developed can be adapted by other facilitators to suit a range of contexts, community member characteristics, and study designs, and the lessons we have learned are broadly applicable to the tasks of collaboratively designing research instruments and analyzing data. At the same time, these materials are not a blueprint that can simply be replicated in other settings. Whenever possible, we drew inspiration from multiple precedents to develop our workshops, but we always tailored exercises and activities provided by other researchers to the HNS’s goals and study communities. Likewise, as the lessons we learned show, iterating upon the workshop plans and improvising in response to resident researchers was crucial to achieving the desired outcomes of the CRD and CDA processes. Thus, while we hope that this article has provided an instructive and practical case study of how to design and facilitate collaborative research design and data analysis within PAR processes, and that the facilitation resources provided as Supplementary Materials prove useful to other researchers, we also acknowledge that even with additional resources at their disposal, facilitators of other PAR processes will nevertheless need to chart their own course based on the communities they are engaging.

## 6. Conclusions

The Healthy Neighborhoods Study Research Consortium used participatory action research to investigate the relationship between urban development and health in nine rapidly-changing neighborhoods in the greater Boston metro area. In response to a shortage of instructional materials in the peer-reviewed literature on how to engage community members in the instrument design and data analysis phases of the study, we developed one series of collaborative research design workshops and two collaborative data analysis workshops to facilitate the engagement of resident researchers in these phases of the study. The 2016 collaborative research design workshops produced a multi-dimensional survey tool, and the 2017 collaborative research design workshops produced a revised survey tool and a semi-structured interview tool. The 2016 and 2018 collaborative data analysis workshops introduced community members to the basics of data analysis and generated interpretations of descriptive statistics and outputs from regression models, structural equation models, and factor analyses as well as new hypotheses for further investigation.

We learned six valuable lessons about workshop design and facilitation in PAR processes. These include: the importance of mechanisms to sustain the continuity of resident researcher participation between workshops, the necessity of a consistent team of facilitators who are able to improvise in response to resident researcher needs, the utility of staggering groups of resident researchers in order to build consensus over time, that building trust between academic researchers and community residents requires making space to discuss power relations and treating points of tension as learning opportunities, and lastly, that a shared commitment to connecting research to action fosters deeper community engagement. If we were to repeat the process, we would also make three important changes. First, we would seek more feedback from colleagues outside the HNS on our workshop materials as they were being developed. Second, we would design a system to evaluate resident researcher experiences during both the CRD and CDA workshops and analyze the evaluation data with a group of resident researchers. Finally, we would plan more opportunities to bring groups of resident researchers from different study communities together during the collaborative research design process to socialize and share ideas and experiences with one another.

## Figures and Tables

**Table 1:** The Healthy Neighborhoods Study (HNS) baseline participatory action research (PAR) process, 2015–2016.

Date	Group 1 (Roxbury, Dorchester, Chelsea)	Group 2 (Everett, Mattapan, Fall River, New Bedford)	Group 3 (Brockton, Lynn)
October 2015	Phase 1: Identifying, recruiting and forming partnerships with community organizations; recruitment of resident researchers	Phase 1: Identifying, recruiting and forming partnerships with community organizations; recruitment of resident researchers	Phase 1: Identifying, recruiting and forming partnerships with community organizations; recruitment of resident researchers
November 2015			
December 2015			
January 2016			
February 2016			
March 2016	Phase 2: Collaborative research design workshops		
April 2016		Phase 2: Collaborative research design workshops	
May 2016			
June 2016	Phase 3: Training on research instruments and study protocol		Phase 2: Collaborative research design workshops
July 2016	Phase 4: Data-gathering	Phase 3: Training on research instruments and study protocol	
August 2016		Phase 4: Data-gathering	
September 2016			Phase 3: Training on research instruments and study protocol
October 2016			Phase 4: Data-gathering
November 2016	Phase 5: Collaborative data analysis workshops	Phase 5: Collaborative data analysis workshops	
December 2016			Phase 5: Collaborative data analysis workshops

**Table 2:** Collaborative research design workshops, 2016.

Workshop	Length	Purpose	Source of Workshop Material
1. Introductions	3 h	Personal introductions, introduction to participatory action research, project description, preliminary discussion of core themes	Original HNS material and [28,29,30,31]
2. Questions	3 h	Generating questions about the relationship between health and place	Original HNS material
3. Methods + Data	3 h	Introduction to research methods and types of data, brainstorm of data that could be collected to help answer questions generated in Workshop 2	Original HNS material and [27]
4. Survey Development	3 h	Review and choose between previously validated measures of variables of interest; begin generation of new measures	Original HNS material
5. Survey Refinement	3 h	Finalize new measures for resident-generated constructs, refine and edit survey in response to contributions of other groups	Original HNS material

HNS: Healthy Neighborhoods Study.

**Table 3:** Collaborative data analysis workshops, 2016.

Workshop Component	Length	Structure	Purpose	Analytical Material
Stage 1: Preparatory workshops in four of nine study communities				
Preparatory Workshop	1.5 h	Stand-alone workshop (4–6 people)	Generate hypothetical models of relations between survey variables	Index cards with all HNS survey variables
Stage 2: “Cluster Workshops”, one in each of three geographic clusters of study communities				
Introduction to Collaborative Data Analysis	25 min	Cluster workshop, full group (12–15 people)	Introductions, goals, agenda, and data review	Descriptive statistics
Analysis of Hypotheses from Preliminary Workshops	30 min	Cluster workshop, small group	Interpret structural equation modelling results	Models from preparatory workshops; Structural equation model
Preliminary Factor Analysis	40 min	Cluster workshop, small group	Interpret preliminary factor analysis results and define underlying constructs	Preliminary factor analysis results for three survey domains
Making Sense of New Themes	30 min	Cluster workshop, small group	Discuss contributions of resident-generated constructs to research goals	Descriptive statistics, factor analysis results
Reflection	50 min	Cluster workshop, full group	Debrief resident researcher experiences of HNS baseline year	N/A

HNS: Healthy Neighborhoods Study; N/A: not applicable.

**Table 4:** Collaborative data analysis workshops, 2018.

Workshop Component	Length	Structure	Purpose	Analytical Material
Introduction, Progress Update, Icebreaker	30 min	Full group	Introductions, goals, agenda, progress update, and build collective energy	N/A
Data Analysis 101	15 min	Full group	Review: what is data, how will we analyze it, and why are we analyzing it together?	N/A
Optional Station: Prioritization	30 min	Small group	Interpretation of descriptive statistics and analytical outputs; guidance on confirmatory factor analysis	Descriptive statistics, regression outputs, preliminary factor analysis results
Optional Station: Ownership of Change	30 min	Small group	Generate hypotheses for quantitative analysis and codes to use in analysis of qualitative interview data	Descriptive statistics
Optional Station: Financial Security	30 min	Small group	Understand how respondents are financially insecure and generate hypotheses about neighborhood-level protective factors	Descriptive statistics
Optional Station: Residential Mobility	30 min	Small group	Discuss just and unjust residential mobility outcomes, differentiate between individual and environmental influences on residential mobility, propose survey revisions	Descriptive statistics
Close out	25 min	Full group	Discuss future steps in analysis process and how residents and organizations will be involved	N/A

## Supplementary Materials

The following are available online at http://www.mdpi.com/1660-4601/16/3/324/s1, S1: Collaborative research design workshop, 2016, facilitator’s guide; S2: Collaborative data analysis workshop, 2018, facilitator’s guide.
