# Supplementary material for: Designing and Facilitating Collaborative Research Design and Data Analysis Workshops: Lessons Learned in the Healthy Neighborhoods Study
Source: Int J Environ Res Public Health. 2019 Jan 24;16(3):324. doi: 10.3390/ijerph16030324 (PMC6388393; doi:10.3390/ijerph16030324)
Supplement: Supplementary file 1 [file ijerph-16-00324-s001.zip › S2 2018 Collaborative Data Analysis Workshops.docx]

**Full Workshop Agenda**

***Healthy Neighborhoods Study – Collaborative Data Analysis, August 2018***

| **Note** |
| --- |
| *This workshop involves four separate Analysis Stations, each corresponding to a domain on the Healthy Neighborhoods Study survey. Each Analysis Station has its own facilitation guide, which begin on page three.* |

| **Time** | **Activity** |
| --- | --- |
| **5m** | **Welcome, Goals, Agenda** |
| **5m** | **Review Progress to Date** |
| **20m** | **Icebreaker Activity** |
| **15m** | **Data Analysis 101** |
|  | Explain what data is and what data analysis is for   - Data are pieces of information about something that can be used in calculating, reasoning or planning   - Give examples of data we have collected - We analyze data to arrive at the findings from our research. Findings are conclusions we make based on the data, and they allow us to tell stories about the issues that we’re investigating together.   - Provide example that illustrates how the same data can be interpreted in different ways.   Explain why we are analyzing data together:   - Reiterate PAR values:   - To center the experience and intelligence of our Resident Researchers.   - To strengthen all of our capacities as activists and researchers in our communities by better equipping us with knowledge about what’s going on and insight into the questions that need to be asked of the powers that be. - Explain relationship between workshop outputs and analyses conducted by academic team   Explain how we will analyze the data together in this workshop   - For each domain, we’ll be reviewing the data that we have collected and begin to interpret it together. As we do so, we will talk about how the patterns we see relate to health, and how those relationships may be different for people with different characteristics (like age or race) and experiences (like discrimination or immigration). - We’ll focus on three types of relationships: Patterns, Cause/Effect, and Inequalities. And at each station you go to, there will be color-coded posters for each. We will try to organize your input according to these three types of relationships. - Each of these categories corresponds to different types of statistical techniques that our team can then do on our end in response to the ideas and guidance you provide us with today. - Reiterate that the big question this workshop is designed to answer is simply: “What do you see and what do you want to know more about”   Describe process of choosing and moving between Analysis Stations |
| **5m** | **Transition to Analysis Station 1** |
| **30m** | **Analysis Station 1** |
| **20m** | **Break** |
| **30m** | **Analysis Station 2** |
| **5m** | **Transition to Analysis Station 3** |
| **30m** | **Analysis Station 3** |
| **5m** | **Transition back to Full Group** |
| **25m** | **Close out** |
|  | Recap the day’s progress and discuss:   - How the analysis process will proceed after the workshop. - How Resident Researchers and Community Partners will be involved in subsequent phases of analysis and in incorporating findings into ongoing community-based work.   Go around the room and ask each participant to share one word about how they feel leaving the workshop today.  Thank staff and participants, and close. |
| **5:45-7:30** | **Dinner + socialize** |

**Financial Security Analysis Station: Facilitator’s Guide**

***Healthy Neighborhoods Study – Collaborative Data Analysis, August 2018***

| **Data Booklet** |
| --- |
| All participants at this station are given a booklet with descriptive statistics on:   - Predictability of monthly income and expenses, broken down by age, self-rated health, and housing tenure. - Difficulty covering monthly expenses, broken down by age, self-rated health, and housing tenure. - The percentage of respondents who came up short on bills in the past 5 years, and what those respondents reported doing in that situation. |

| **Time** | **Facilitation** |
| --- | --- |
| **2-3m** | **Introduction and Framing** |
|  | Introduce the topic of financial security and offer a working definition.  Reiterate where financial security fits within our broader theory of change.  Specify three guiding questions for the discussion:   - What are the different factors internal and external to a person that may cause financial security, and how are they related? - How can neighborhoods protect people from financial insecurity and/or its negative consequences? - What aspects of neighborhoods exacerbate financial insecurity and/or make its consequences more significant?   Specify which survey measures the data being analyzed at this station comes from.  Reiterate that responses will be organized three categories of relationships to explore in further analysis: Patterns, Inequalities, and Cause/Effect |
| **25m** | **Analysis** |
|  | 1. Describe the contents of the Data Booklet 2. Ask if there are any clarifying questions about what analysis was performed or the materials, answer as appropriate. 3. Go-round: What’s one data point that jumps out at you and why?    1. For each one: What do you think are important individual-level factors (things about the person) that you think would play an important role in that outcome, and what are the important neighborhood-level factors (things about the neighborhood/environment) ? 4. **Open Discussion**: Based on the discussion so far, what ideas come to you about *how* people are financially insecure? Are there different ways of being financially insecure? What’s different about them and what’s important about these differences?   [Objective: Understand different types of financial security and the elements of each]   1. **Open Discussion:** For a person (hypothetical or not) in one of these different types of financial insecurity, what types of neighborhood-level changes do you think would *prevent* these types of insecurity and/or lessen their negative consequences?   [Objective: Understand protective factors] |
| **2-3m** | **Wrap-up + next steps** |
|  | - Recap major themes of discussion - Indicate how the academic team will build on this analysis moving forward - Thank participants |

**Ownership of Change Analysis Station: Facilitator’s Guide**

***Healthy Neighborhoods Study – Collaborative Data Analysis, August 2018***

| **Data Booklet** |
| --- |
| All participants at this station are given a booklet with descriptive statistics on responses to the Ownership of Change measure. |

| **Time** | **Facilitation** |
| --- | --- |
| **2-3m** | **Introduction and framing** |
|  | Ownership of Change is one of the parts of the survey that was designed from scratch by Resident Researchers, which means we’re still in the process of figuring out how to interpret data from this measure.  Ground this theme in the Theory of Change.  Specify measures of Ownership of Change on the survey.  Specify objectives for this station:   1. Generate hypotheses together about what may be happening to produce the results we see. 2. Talk about how to use the narrative data from the qualitative interviews to learn more about what Ownership of Change means.   Reiterate focus on three types of relationships to explore in greater depth: Cause-Effect, Inequalities, and Patterns. |
| **25m** | **Analysis** |
|  | 1. Describe the contents of the Data Booklet 2. Ask if there are any clarifying questions about what analysis was performed or the materials, answer as appropriate. 3. Go-round: what’s one data point that jumps out at you and why?    1. Facilitator probes each participant with focus on cause/effect relationships. For example: What’s one idea you have about why that data point is what it is? What could be causing that outcome? What is this an effect of? What might this data point be causing? What might happen as a result? 4. Zoom out: As you’ve been listening to each other’s reactions, what thoughts have you had about bigger patterns or questions that come out of this data?    1. Potential prompt: what do you think is happening with [type of change] or [type of ownership]?   [Objective: hypothesis generation]   1. Looking ahead: As we’re analyzing what people say in interviews about the theme of ownership of change, what should we be investigating?    1. Ask for keywords and a justification    2. Additional probes: What might people be telling us in interviews that can help us understand these numbers more? What part of the story about peoples’ sense of ownership over local changes are these numbers not telling us? What dimensions of Ownership of Change need to be explained via voice/narrative/experience instead of quantitative data?   [Objective: Generate ideas for qualitative codes] |
| **2-3m** | **Wrap-up + next steps** |
|  | - Recap major themes of discussion - Indicate how the academic team will build on this analysis moving forward - Thank participants |

**Prioritization Analysis Station: Facilitator’s Guide**

***Healthy Neighborhoods Study – Collaborative Data Analysis, August 2018***

| **Data Booklet** |
| --- |
| All participants at this station are given a booklet with:   - Descriptive statistics on level of importance assigned to each priority on the Prioritization inventory - Descriptive statistics on priority fulfillment rates for each priority on the Prioritization inventory - Preliminary associations between priority fulfillment and health, mental health and happiness outcomes   The results of an exploratory factor analysis were visually represented using flipcharts posted on the walls of the room, but were not provided in the data booklet. |

| **Time** | **Facilitation** |
| --- | --- |
| **5m** | **Introduction and Framing** |
|  | This station is focused on the Prioritization section of the survey, which helps us understand a person’s most important priorities and the extent to which they are able to fulfill them. This section, like Ownership of Change, is one of the parts of the survey that is a unique contribution of Resident Researchers and the PAR process to the research field.  Discuss where Prioritization fits within the broader Theory of Change  Discuss progress to date analyzing data in this domain, with specific reference to hypotheses generated by Resident Researchers for the 2016 Collaborative Data Analysis workshop. |
| **20m** | **Analysis** |
|  | 1. Describe the contents of the Data Booklet 2. Ask if there are any clarifying questions about what analysis was performed or the materials, answer as appropriate. 3. Go-round: Looking at the descriptive statistics, what’s one data point that jumps out at you and why? Facilitator probe focuses on cause/effect relationships, for example:    - - What’s one idea you have about why that data point is what it is? What could be causing that outcome? What is this an effect of?      - What might this data point be causing? What might happen as a result? 4. Turn to preliminary associations between priorities and health outcomes. Ask participants which associations they have a reaction to (surprise, confusion, validation, etc), and why. Ask participants to generate hypotheses about why certain associations are or are not present. 5. Explain that an exploratory factor analysis is used to explore the underlying structure of what is being measured. A factor analysis tells us which items “travel together” – for example, if people who prioritize A also tend to prioritize B and C, and people who prioritize D also tend to prioritize E and F, ABC would be a factor, and DEF would be a factor. The challenge then becomes how to interpret what it means that A, B and C “travel together” 6. Explain results of exploratory factor analysis, and specify what needs to be resolved in order to move to confirmatory factor analysis. Solicit reactions to preliminary factor analysis and suggestions for factors to specify in confirmatory factor analysis.   [Display flipchart of exploratory factor analysis results] |
| **2-3m** | **Wrap-up + next steps** |
|  | - Recap major themes of discussion - Indicate how the academic team will build on this analysis moving forward - Thank participants |

**Residential Mobility Analysis Station: Facilitator’s Guide**

***Healthy Neighborhoods Study – Collaborative Data Analysis, August 2018***

| **Data Booklet** |
| --- |
| All participants at this station are given a booklet with:   - Descriptive statistics on reasons for recent moves and anticipated moves |

| **Time** | **Facilitation** |
| --- | --- |
| **5m** | **Introduction and Framing** |
|  | Describe the theme of this station: This station is focused on what we call “residential mobility,” which refers to patterns of moving (or staying) in homes over time and space, both in individual people’s lives and at a population level. Forced displacement is one of many types of residential mobility that our research captures; others might be being stuck in one place, or choosing to move to be closer to family, for example.  Describe how Residential Mobility figures in to the Theory of Change.  Describe the purpose of this station:   1. First, we’re going to talk about what “good” residential mobility outcomes are, what people want or hope for, and what we think is just; this will then help us point out where the problem areas really are. 2. Second, we’re going to try to tease apart what matters in terms of the person themselves and their life, versus what matters in terms of the person’s environment, in producing positive and negative residential mobility outcomes. |
| **20m** | **Analysis** |
|  | 1. Describe advance analysis, and answer any clarifying questions about what is provided in the data booklet 2. Facilitate open discussion based on this prompt: One way to approach the question of residential mobility is from the perspective of justice. While we can probably all agree that forced displacement is unjust, we might also agree that being stuck somewhere and being unable to move if you want or need to is also an unjust housing situation. So what’s the sweet spot in the middle? What does “just” residential mobility look like? 3. Solicit input: Ask participants to look at data and propose examples of Just (eg. moving to a good school district) and Unjust (eg. eviction) residential mobility experiences. For each outcome proposed, ask what role the person and their environment each play in producing this outcome   [Write responses on flipcharts, one for “Just” outcomes and one for “Unjust” outcomes]   1. Pick one outcome at a time, and ask participants to discuss what they would expect in terms of the chains of consequences in people’s lives, social networks, and communities when these outcomes occur. Go through as many outcomes as you can in the time available, alternating between “Just” and “Unjust” outcomes as listed in the previous step.   [Draw chains of consequences on a “Consequences” flipchart] |
| **2-3m** | **Wrap-up + next steps** |
|  | - Recap major themes of discussion - Indicate how the academic team will build on this analysis moving forward - Thank participants |
