# Supplementary material for: Designing and Facilitating Collaborative Research Design and Data Analysis Workshops: Lessons Learned in the Healthy Neighborhoods Study
Source: Int J Environ Res Public Health. 2019 Jan 24;16(3):324. doi: 10.3390/ijerph16030324 (PMC6388393; doi:10.3390/ijerph16030324)
Supplement: Supplementary file 1 [file ijerph-16-00324-s001.zip › S1 2016 Collaborative Research Design Workshops.docx]

**APPENDIX 1**

**Phase 2, 2016: Knowledge Exchange and Instrument Design Workshops**

**Collaborative Research Design Workshop 1: Introduction and Goals**

***Healthy Neighborhoods Study – Planning Phase (2016)***

| **Desired Outcomes** |
| --- |
| - Agreement to continue as a group with values alignment towards common goal - Understanding of PAR and scope of Healthy Neighborhoods Study - Begin collective conversation about relationship between health and place |

| **Agenda Overview (3h)** |
| --- |
| 1. Open up and icebreakers (15m) 2. Introduction to Healthy Neighborhoods Study (30m) 3. Reactions (15m) 4. Power and values in knowledge creation (40m) 5. BREAK (15m) 6. Health and Place (50m) 7. Homework and wrap up (10m) |

| **Time** | **Activity** |
| --- | --- |
| 15m | **Introductions and Icebreaker Activity** |
| 30m | **Introduction to Healthy Neighborhoods Study**   1. Provide history of HNS: how and why it was established; who is funding the work; what partners are involved so far; what the objectives of these partners are and what progress has been made towards fulfilling their objectives; why and how community participation was prioritized; the process by which participating community organizations and residents were recruited; Resident Researcher role, responsibilities and compensation; and how academic and institutional partners view Participatory Action Research, and what they hope to achieve through a participatory approach. 2. Describe process goal established by academic and institutional partners: academic and community partners working equitably and collaboratively to answer mutually defined research questions using mutually agreed-upon research methods, data analytics, and dissemination strategies. 3. Share a proposal for the next six workshops:    1. Develop research questions, tools, process    2. Develop research skills so that everyone is equipped to use the tools 4. Discuss longer-term goals established by academic and institutional partners:    1. Shared decision-making across research process    2. Development of sustainable community research infrastructure to support future years of the study    3. Translate research findings into actions to improve community health and wellbeing |
| 15m | **Discussion**   1. Potential facilitator prompts include: What are your reactions (to the history, goals, structure, other issues)? What do you (individually, as members of this group, as members of the community) hope to get out of participating? What experiences, knowledge and skills do each of you bring to the project? |
| 45m | **Power and Values in Knowledge Creation**   1. The goal of this section is to explore: different ways of knowing; knowledge production; and “research justice” 2. Introduce different types of knowledge: Cultural & Spiritual, Experiential, Institutional, Embodied. Present in quadruple venn diagram.**^[[1]](#footnote-1)^**    1. Cultural & Spiritual: practices and wisdom passed down from our community (elders, neighbors, family, friends)    2. Experiential: lived experience, what we learn and know from living and dealing with issues that impact our lives    3. Institutional: published facts and data produced by “professionals” usually from outside the community    4. Embodied: physical and mental feelings, senses and processes 3. **Discussion:** How are different types of knowledge respected in your community? Solicit examples of how different types of knowledge have been received differently. 4. Present metaphors for understanding our work together    1. “Building a microphone”: the research tools we design together will be like building a microphone to amplify the voices of residents and bring their experiences into the conversation about urban development and community health    2. “Making fruit salad”: Arriving at the right mix of data representing different types of knowledge is like making fruit salad – all the ingredients taste best together, and we can see when institutions or people in positions of power “pick out the grapes” or “only eat the melon.” 5. **Exercise:** Who’s got the power?^[[2]](#footnote-2)^ 6. **Exercise:** The Knowledge Factory^[[3]](#footnote-3)^ |
| 15m | **BREAK** |
| 50m | **Exploring the relationship between health and place**   1. This project asks us to think about the places we call home and how they influence our health, particularly as these places change 2. Let’s talk about place:    1. **Discussion**: How would you describe your community to an outsider?    2. **Discussion:** What is your vision for the future of your community? 3. Transition to discussion about health    1. Facilitator introduces       1. definitions of health from WHO^[[4]](#footnote-4)^ and Huber et al^[[5]](#footnote-5)^       2. “social determinants” and health promotion strategies;^[[6]](#footnote-6)^       3. examples of influencing discussions of housing policy/community and economic development    2. Facilitator solicits reactions    3. **Discussion:** health and place in your community. Facilitator probes for discussion of:       1. Health disparities, and theories Resident Researchers have to explain who is healthy, who is sick, and why       2. Role of neighborhood in protecting or harming health       3. How could urban development affect health, both positively and negatively?       4. What would the development need to be like to be good for your community’s health?       5. What and who in your community helps everyone stay healthy?       6. What community-level problems would you solve to promote health?       7. What would a healthy neighborhood look like?       8. How can the people and structures in your environment create health rather than merely prevent disease?       9. How do healthy versus sicker people experience life in your community? |
| 5m | **Schedule and preview next session** |
| 5m | **Homework for next session (see Handout 1, below)**   1. Health determinant research question exercise 2. Brainstorm principles and procedures that you would like our group to agree to. We’ll discuss these further and reach agreement in our next session |

**Collaborative Research Design Workshop 2: Research Questions**

***Healthy Neighborhoods Study – Planning Phase (2016)***

| **Desired Outcomes** |
| --- |
| - Generate preliminary research questions that will serve as a foundation for identifying data-gathering needs - Continue to develop teamwork and collaboration among the group |

| **Agenda Overview (3h)** |
| --- |
| 1. Check in and recap (20m) 2. Affirm core principles and operating norms (20m) 3. Homework share-back (20m) 4. Homework debrief and brainstorm patterns and themes (35m) 5. BREAK (15m) 6. Research question generation (60m) 7. Homework and wrap up (10m) |

| **Time** | **Activity** |
| --- | --- |
| 20m | **Check-in and recap**   1. **Go-round:** Icebreaker activity 2. Summarize main points and milestones from last week 3. Introduce goals for this session |
| 20m | **Affirm core principles and group operating norms**   1. **Go-round:** Each participant shares “principles and procedures” proposals from homework exercise 2. Facilitate agreement on group operating norms (drawing, if necessary, on a list of examples) |
| 20m | **Homework share-back**   1. **Go-round:** Participants take turns sharing observations from homework. Facilitators may seek reactions from other participants. One facilitator should take notes on large paper or a whiteboard so that all participants can keep track. |
| 35m | **Debrief and brainstorm patterns and themes**   1. **Discussion:** Facilitate an open discussion reflecting on the group’s outputs from the homework activity with the intention of identifying key patterns and themes concerning the relationship between neighborhood development and health that may merit further inquiry through data collection and analysis. Be as broad and deep as possible. |
| 15m | **BREAK** |
| 60m | **Research question generation**   1. Building off of **Homework 1** facilitate a discussion to identify core research questions about the relationship between health and development that will set us up to think about sources of information/data in the next session. Use guiding questions, for example:    1. Who might be affected by transit-oriented development and how? Will different groups of people be affected differently?    2. If you were responsible for planning new developments so that they had a positive impact on neighborhood health, what changes would you prioritize?    3. What information is important for the community to have access to and/or ownership over in order to advocate for healthy development decisions?    4. When you think about your own vision of what a healthy community is, what questions come to mind about how to get from here to there? What would you need to know to help develop a roadmap? 2. Present research questions of interest to academic, non-profit and public sector members of the Consortium. Discuss: How do these relate to our research priorities? Do they feel relevant and important? Are there ways you might change their focus to make the research more valuable for your community? 3. Wrap up by consolidating core themes and questions to set foundation for next session’s discussion on the data sources that will help answer these questions. |
| 10m | **Wrap-up and homework**   1. Plus/Delta on group process during this meeting. (Pluses are things that worked well, and Deltas are things that you would change for next time). 2. Introduce “data brainstorm” homework **(Handout 2)** |

**Collaborative Research Design Workshop 3: Data**

***Healthy Neighborhoods Study – Planning Phase (2016)***

| **Desired Outcomes** |
| --- |
| - Identify data that will help us answer questions identified in Workshop 2 - Develop a shared understanding of how to employ different research methods - Continue to develop teamwork and collaboration among the group |

| **Agenda Overview (3h)** |
| --- |
| 1. Check in and recap (15m) 2. Homework debrief (45m) 3. Introduce Methods, pt. 1: Interviews and surveys (30m) 4. BREAK (15m) 5. Introduce Methods, pt. 2: Focus groups and asset mapping (30m) 6. Matching data with methods (45m) 7. Wrap-up and homework (10m) |

| **Time** | **Activity** |
| --- | --- |
| 15m | **Check-in and recap**   1. Fun, quick icebreaker (eg. everyone describes the superpower they would have as a comic book hero) 2. Summarize main points and milestones from last week 3. Introduce goals for this session |
| 45m | **Homework debrief**   1. In advance, write each question generated in Workshop 2 on a large piece of paper and post them on the wall around the room. Give participants post-it notes and ask them to write each piece of information/data they generated during their homework on a post-it and then move around the room posting them on the paper for the research question they think the data will help answer. 2. Then, ask everyone to walk around and observe what others posted, and add their own ideas with additional/remaining post-it notes. 3. Re-group, and together, consider each question on its own. Review the different types of information/data suggested to help answer the question with a focus on:    1. Synthesizing similar suggestions    2. Being as specific as possible    3. Clarity on why the data suggested will help answer the question asked. 4. Zoom back out to the set of questions once more as a whole, and as a group reflect on the following prompts:    1. Imagine what you care most about in your life, what causes you the most joy and worry, etc. Are these questions relevant? Is there an angle we’re forgetting about?    2. Are we adequately accounting for the concerns of fellow community members not in this room (and/or not represented by the people in this room)?    3. Do we account for the concerns and priorities of other HNS members not in the room today?   **If possible, take a photograph when finished, as the post-its will be moved to different flipcharts in a subsequent activity.* |
| 30m | **Methods Introduction: Interviews and Surveys (15m +15m)**   1. Spend 15 minutes each on Interviews and Surveys. For each method:    1. Describe aims and objectives of method^[[7]](#footnote-7)^    2. Describe an impact-oriented example relevant to the community or major research themes.    3. Facilitator-led improvised prototyping based on Research Questions and Homework Gallery. For 3-4 minutes, a facilitator demonstrates the method in action. The group then discusses what types of data this method lends itself to gathering. |
| 15m | **Break** |
| 30m | **Methods Introduction: Focus Groups and Asset Mapping (15m +15m)**   1. Spend 15 minutes each on Focus Groups and Asset Mapping. For each method:    1. Describe aims and objectives of method^[[8]](#footnote-8)^    2. Describe an impact-oriented example relevant to the community or major research themes.    3. Facilitator-led improvised prototyping based on Research Questions and Homework Gallery. For 3-4 minutes, a facilitator demonstrates the method in action. The group then discusses what types of data this method lends itself to gathering. |
| 35m | **Matching data with methods**   1. Make a flipchart for each method, and post them up on the wall. 2. Return to the homework debrief, and proceeding question-by question, take each data need and discuss which method is best suited to gathering the needed data. Once the group has reached consensus, move that post-it from the question flipchart to the method flipchart. If the group agrees that a specific data need should be addressed through multiple methods, create a second post-it for that data need and post it on both methods. 3. By the end of the exercise, the group should have a shared sense of which methods they think are most appropriate to gather the data they have deemed most important to answer the questions that matter to them. 4. Depending on your study and capacity, discuss which method(s) to prioritize developing first as the group moves in to the tool-building stage. |
| 10m | **Wrap-up and debrief**   1. Plus/Delta on group process during this meeting. (Pluses are things that worked well, and Deltas are things that you would change for next time). 2. Introduce homework: Facilitators distribute potential components of data-gathering tools between this workshop and the next. Resident Researchers read through them and practice using them on family or friends. As they do so, they take notes on how the questions feel to ask and answer, and how/whether the data they generate helps to answer the group’s research questions as articulated in the workshop. |

**Collaborative Research Design Workshop 4: Survey Development 1**

***Healthy Neighborhoods Study – Planning Phase (2016)***

| **Note** |
| --- |
| *Please note that, at the end of Workshop 3, HNS Resident Researchers agreed to prioritize developing a community survey in the first year of the project, and then building a semi-structured interview tool to gather qualitative data after piloting the survey in the baseline year. As a result, this workshop is oriented towards survey development. Depending on the priorities of your group, you may need to adapt the agenda to align with the tools you have chosen to develop.*  *Between Workshop 3 and Workshop 4, the facilitators compiled a wide range of previously validated measures of variables of concern to the Resident Researchers. These measures were distributed to Resident Researchers as part of their homework* |

| **Desired Outcomes** |
| --- |
| - Begin to draft survey sections - Note questions and topics that should subsequently be covered in interviews and focus groups |

| **Agenda Overview (3h)** |
| --- |
| 1. Check in and recap (15m) 2. Focusing reflection (25m) 3. Survey tool refinement, Part 1 (50m) 4. Break (15m) 5. Survey tool refinement, Part 2 (55m) 6. Wrap up (20m) |

| **Time** | **Activity** |
| --- | --- |
| 15m | **Check-in and Recap**   1. Icebreaker activity 2. Recap where the group left off last time, homework assignment, and fill the group in on the progress of other community teams working in parallel. 3. Review objectives for the workshop 4. Describe how the facilitators compiled previously validated measures (and what “previously validated” means) and how they’ve been used. |
| 25m | **Focusing reflection**   1. Give participants 5-10 minutes to fill out worksheet **(Handout 3)** 2. Ask for volunteers to share some of their responses, and facilitate a short group discussion about what emerges, with the aim of preparing the group to make decisions about the content of the survey |
| 50m | **Survey tool refinement, Part 1**   1. Proceeding topic by topic, facilitators should introduce the options for previously validated measures that were circulated among the group. For each topic:    1. Facilitators should solicit the group’s reactions to practicing with these measures on their friends/family    2. The group should discuss which options are most effective at gathering the type of data they prioritized in Workshop 3.    3. The group should reach agreement about which measure they would prefer to use, as well as a second choice (in case other groups overwhelmingly prefer another option). The group should specify whether they would like to use the measure as-is or with edits (and if so, what those edits might be). Alternatively, the group may conclude that that topic requires building a new measure from scratch as a group.    4. Note in a “parking lot” whether there are aspects of this topic that would be better addressed through other methods (eg. interviews) in subsequent phases of the study. |
| 15m | **Break** |
| 55m | **Survey tool refinement, Part 2**  1. Continue process described in Part 1 until all topics have been covered. |
| 20m | **Wrap-up and Homework**   1. Review decisions/outcomes of this workshop 2. Present next step:    1. Facilitators draft full survey synthesizing input from all groups, and distribute back to Resident Researchers and community partner organizations in advance of Workshop 5 3. Closing comment/reflection from each person 4. Homework: Facilitators will share draft survey by email. Homework is to run it by a couple friends/family members and explain what it’s for. Make notes about what works well and what may need further discussions or edits/modifications. Discuss with the friends/family you practice on whether they think the survey will get the information that the community needs. |

**Collaborative Research Design Workshop 5: Survey Development 2**

***Healthy Neighborhoods Study – Planning Phase (2016)***

| **Note** |
| --- |
| *Please note that, at the end of workshop three, HNS Resident Researchers agreed to prioritize developing a community survey in the first year of the project, and then building a semi-structured interview tool to gather qualitative data after piloting the survey in the baseline year. As a result, this workshop is oriented towards survey development. Depending on the priorities of your group, you may need to adapt the agenda to align with the tools you have chosen to develop.*  *Between Workshop 4 and Workshop 5, the facilitators assembled a draft version of the full survey that incorporates (and in some cases synthesizes) feedback from all groups. The draft survey was distributed to Resident Researchers to use as part of the homework from Workshop 4.* |

| **Desired Outcomes** |
| --- |
| - Begin to draft survey sections - Note questions and topics that should subsequently be covered in interviews and focus groups |

| **Agenda Overview (3h)** |
| --- |
| 1. Check in and recap (15m) 2. Survey practice (30m) 3. Survey practice debrief (30m) 4. Break (15m) 5. Survey edits and refinement (60m) 6. Survey logistics (20m) 7. Wrap-up (10m) |

| **Time** | **Activity** |
| --- | --- |
| 15m | **Check in and recap**   1. Icebreaker activity 2. Recap outcomes of previous workshop and relevant progress updates from other groups that have met in the interim 3. Describe facilitators’ process assembling draft of full survey after previous workshop |
| 30m | **Survey practice**   1. In pairs, take turns surveying one another. As participants progress, they should discuss how the questions feel to ask and to answer, note their reactions and any suggested edits, questions, or other ideas for improving the instrument. Facilitators observe. |
| 30m | **Survey practice debrief**   1. Proceed through the survey section by section, gathering feedback/edits/suggestions from all pairs and listing them on flipchart paper so the whole group can see. If multiple groups practiced the same section(s), note whether there were areas of agreement/disagreement in their remarks.    1. The purpose of this activity is to surface suggestions, not make decisions. Decisions will be discussed after the break. |
| 15m | **Break** |
| 60m | **Survey edits and refinement**   1. Return to list from survey practice debrief, and resolve as many issues as possible. Be sure to note that these decisions are not final until they are also agreed upon by other groups, and that if there is significant disagreement, we will have a follow-up discussion to reconcile. Likewise, facilitators should note which edits the group feels strongly about and which they feel more flexible about, so that it’s clear how much the group is willing to accommodate other ideas from other groups. 2. If possible, try to identify items that can be cut with limited consequence; indicate that, if there is consensus among groups on cutting something, the facilitators will remove the item. |
| 20m | **Survey logistics**   1. Briefly discuss the logistics of surveying (eg. what should information cards for respondents say?) and address any Resident Researcher concerns |
| 10m | **Wrap-up**   1. Recap progress made 2. Plus/delta evaluation process and teamwork |

**Handout 1: Workshop 1 Homework**

**Journaling**: What and who influences your health (for better or worse) in your daily life? Take ten minutes to feel your current surroundings and remember your past day or week. What-- currently, in the past, or in the future-- makes you feel good? What makes you feel worse? Please be as specific as you comfortably can. Try to complete this exercise at least three times before our next meeting, choosing different times and places to sit and write.

Try to note: *people, places, objects/material goods, inner dialogue, internal or external pressures, sounds, sights, smells, absences, expectations, perceptions, times you laughed, times you tensed up, etc.*

Do this at several places/points: *at home, on your commute, during leisure time, in a public space, etc.*

| HOME | COMMUTE |
| --- | --- |
| LEISURE | PUBLIC SPACE |

**Brainstorming:** Principles and procedures that our group should commit to. There are some examples in your folder.

Principles:

______________________________________________________________________________________________________________________________________________________________________________________________________________________________________________________________________________________________________________________________________________________________________________________________________________________________________________________________________________________________________________________________________________________________________________________________________________________________________________________________________________________________________________________________________________________________________________________________________________________________________________________________________________________________________________________________________________________________________________________________________________________________________________________

Procedures:

______________________________________________________________________________________________________________________________________________________________________________________________________________________________________________________________________________________________________________________________________________________________________________________________________________________________________________________________________________________________________________________________________________________________________________________________________________________________________________________________________________________________________________________________________________________________________________________________________________________________________________________________________________________________________________________________________________________________________________________________________________________________________________________

**Handout 2: Workshop 2 Homework**

Explain this project to at least three friends or family members. Tell them the research questions that your group discussed, and ask them for their feedback about the following questions:

1. Do they make sense in real life?

______________________________________________________________________________________________________________________________________________________________________________________________________________________________________________________________________________________________________________________________________________________________________________________________________

____________________________________________________________________________________________________________________________________________________________

2. Are there angles that we’ve overlooked?

______________________________________________________________________________________________________________________________________________________________________________________________________________________________________________________________________________________________________________________________________________________________________________________________________

______________________________________________________________________________

3. Do these questions feel important to you, and why?

______________________________________________________________________________________________________________________________________________________________________________________________________________________________________________________________________________________________________________________________________________________________________________________________________

__________________________________________________________________________________________________________________________________________________________________________________________________________________________________________

4. And, most importantly: What information (data) might help us find an answer to this question?

______________________________________________________________________________________________________________________________________________________________________________________________________________________________________________________________________________________________________________________________________________________________________________________________________

__________________________________________________________________________________________________________________________________________________________________________________________________________________________________________

**Handout 3: Workshop 4 Worksheet**

*Reflect on the following questions and jot down a few of your thoughts in response to each.*

1. What do you hope this research will do for your neighborhood in 2 years? How about in 10 years? What would our survey and interviews have to be like to help us get there?

___________________________________________________________________________

___________________________________________________________________________

___________________________________________________________________________

___________________________________________________________________________

___________________________________________________________________________

___________________________________________________________________________

___________________________________________________________________________

___________________________________________________________________________

2. What is something that you want a real estate developer or funder to learn from this research? How do you think it would change the way they intervene in your community?

___________________________________________________________________________

___________________________________________________________________________

___________________________________________________________________________

___________________________________________________________________________

___________________________________________________________________________

___________________________________________________________________________

___________________________________________________________________________

___________________________________________________________________________

3. What do you want residents of your neighborhood to learn from your research? Why?

___________________________________________________________________________

___________________________________________________________________________

___________________________________________________________________________

___________________________________________________________________________

___________________________________________________________________________

___________________________________________________________________________

___________________________________________________________________________

___________________________________________________________________________

**Works Cited**

DataCenter. (2013). *An Introduction to Research Justice*. Oakland, CA: DataCenter.

Huber, M., Knottnerus, J. A., Green, L., Horst, H. van der, Jadad, A. R., Kromhout, D., … Smid, H. (2011). How should we define health? *BMJ*, *343*, d4163. https://doi.org/10.1136/bmj.d4163

McGinnis, J. M., Williams-Russo, P., & Knickman, J. R. (2002). The Case For More Active Policy Attention To Health Promotion. *Health Affairs*, *21*(2), 78–93. https://doi.org/10.1377/hlthaff.21.2.78

Urban Justice Center. (n.d.). *Research for Organizing: A Toolkit for Participatory Action Research*.

World Health Organization. (n.d.). WHO | Frequently asked questions. Retrieved August 30, 2018, from http://www.who.int/suggestions/faq/en/

1. Adadpted from DataCenter (2013) [↑](#footnote-ref-1)
2. DataCenter (2013) [↑](#footnote-ref-2)
3. DataCenter (2013) [↑](#footnote-ref-3)
4. (World Health Organization, n.d.) [↑](#footnote-ref-4)
5. (Huber et al., 2011) [↑](#footnote-ref-5)
6. Drawing on McGinnis et al (2002) [↑](#footnote-ref-6)
7. Drawing from Urban Justice Center (n.d., pp. 34–88) [↑](#footnote-ref-7)
8. Drawing from Urban Justice Center (n.d., pp. 34–88) [↑](#footnote-ref-8)
